# Survival benefit of primary prevention implantable cardioverter‐defibrillator/cardiac resynchronization therapy with a defibrillator: Analysis of the Japan cardiac device treatment registry and Japanese cardiac registry of heart failure in cardiology

**DOI:** 10.1002/joa3.70084

**Published:** 2025-05-12

**Authors:** Hisashi Yokoshiki, Masaya Watanabe, Sanae Hamaguchi, Hiroyuki Tsutsui, Akihiko Shimizu, Takeshi Mitsuhashi, Kohei Ishibashi, Tomoyuki Kabutoya, Yasuhiro Yoshiga, Yusuke Kondo, Taro Temma, Masahiko Takagi, Hiroshi Tada

**Affiliations:** ^1^ Department of Cardiovascular Medicine Sapporo City General Hospital Sapporo Japan; ^2^ Department of Cardiovascular Medicine Hokko Memorial Hospital Sapporo Japan; ^3^ School of Medicine and Graduate School International University of Health and Welfare Otawara Japan; ^4^ UBE Kohsan Central Hospital Ube Japan; ^5^ Department of Cardiovascular Medicine Hoshi General Hospital Koriyama Japan; ^6^ Department of Cardiovascular Medicine National Cerebral and Cardiovascular Center Suita Japan; ^7^ Division of Cardiovascular Medicine, Department of Medicine Jichi Medical University School of Medicine Shimotsuke Japan; ^8^ Division of Cardiology, Department of Medicine and Clinical Science Yamaguchi University Graduate School of Medicine Ube Japan; ^9^ Department of Cardiovascular Medicine Chiba University Graduate School of Medicine Chiba Japan; ^10^ Department of Cardiovascular Medicine Hokkaido University Hospital Sapporo Japan; ^11^ Division of Cardiac Arrhythmia Kansai Medical University Medical Centre Moriguchi Japan; ^12^ Department of Cardiovascular Medicine, Faculty of Medical Sciences University of Fukui Fukui Japan

**Keywords:** cardiac resynchronization therapy with a defibrillator, heart failure with reduced ejection fraction, implantable cardioverter‐defibrillator, Japan cardiac device treatment registry database, Japanese cardiac registry of heart failure in cardiology

## Abstract

**Background:**

Evidence supporting the benefit from primary prevention implantable cardioverter‐defibrillator (ICD)/cardiac resynchronization therapy with a defibrillator (CRT‐D) for heart failure with reduced ejection fraction (HFrEF) is scarce in real‐world settings.

**Methods:**

We analyzed propensity score matched cohorts of patients eligible for Sudden Cardiac Death in Heart Failure Trial (SCD‐HeFT) from Japan cardiac device treatment registry (JCDTR) and Japanese Cardiac Registry of Heart Failure in Cardiology (JCARE‐CARD). The former served as the defibrillator therapy group and the latter as the conventional therapy group.

**Results:**

During an average follow‐up of 24 months, death occurred in 35 of 285 patients (12%) with defibrillator therapy and 65 of 285 patients (23%) with conventional therapy. Adjusted hazard ratios of all‐cause death, sudden death, heart failure death, and noncardiac death in defibrillator versus conventional therapy were 0.616 (95% confidence interval [CI]: 0.402–0.943, *p* = 0.026), 0.274 (95% CI: 0.103–0.731, *p* = 0.0097), 0.362 (95% CI: 0.172–0.764, *p* = 0.0077) and 1.45 (95% CI: 0.711–2.949, *p* = 0.31). After accounting for death without appropriate defibrillator therapy as a competing risk, the cumulative incidence of first appropriate defibrillator therapy in the defibrillator therapy group was nearly identical to that of all‐cause death in the conventional therapy group. Subgroup analyses indicated a lack of defibrillator benefit in patients with hypertension (*p* = 0.01 for interaction).

**Conclusions:**

Primary prevention ICD/CRT‐D reduced the risk of all‐cause mortality of patients with HFrEF eligible for SCD‐HeFT compared to conventional therapy in the real‐world cohort.

## Graphical abstract

Among propensity‐matched cohorts eligible for SCD‐HeFT, primary prevention ICD/CRT‐D reduced the risk of all‐cause mortality with an adjusted hazard ratio of 0.616 (*p* = 0.026). The cumulative incidence of first appropriate therapy in the defibrillator therapy group was nearly identical to that of all‐cause death in the conventional therapy group.

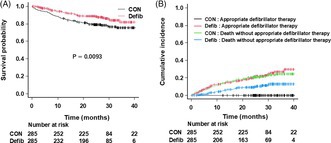

## 1 INTRODUCTION

Development of the implantable cardioverter‐defibrillator (ICD)^1^, ^2^, ^3^ has undoubtedly contributed to the major advance in cardiovascular medicine, despite initial criticism.^4^ Transvenous lead systems yielded widespread use of ICDs, which reduced mortality in patients with a history of sustained ventricular tachycardia (VT) or fibrillation (VF).^5^ Thereafter, ICDs proved to be effective for primary prevention of sudden cardiac death in ischemic patients with left ventricular ejection fraction (LVEF) ≦30% (Multicenter Automatic Defibrillator Implantation Trial [MADIT] II)^6^ and in heart failure patients with LVEF ≦35% and New York Heart Association (NYHA) classification II or III (Sudden Cardiac Death in Heart Failure Trial [SCD‐HeFT]).^7^

At the time around completion of SCD‐HeFT, cardiac resynchronization therapy with (CRT‐D) or without (CRT‐P) a defibrillator reduced mortality in symptomatic heart failure patients with LVEF of 35% or less and QRS width of 120 msec or more.^8^, ^9^ Among de novo implantation, the proportion of primary prevention CRT‐D is about 70%, whereas that of primary prevention ICD is less than 30% in Japan.^10^ In this regard, the defibrillator therapy including both ICDs and CRT‐Ds is likely to show the real‐world effectiveness for primary prevention of sudden cardiac death in patients with heart failure with reduced ejection fraction (HFrEF). To this end, we analyzed outcomes of heart failure patients enrolled in the Japan Cardiac Device Treatment Registry (JCDTR)^11^ for defibrillator therapy and those enrolled in the Japanese Cardiac Registry of Heart Failure in Cardiology (JCARE‐CARD)^12^, ^13^, ^14^ for conventional therapy.

## 2 METHODS

### 2.1 Study population

The study cohort consists of (a) 746 patients with LVEF ≦35% and an ICD or CRT‐D for primary prevention of sudden cardiac death enrolled in the Japan Cardiac Device Treatment Registry (JCDTR) with an implantation date from January 2011 to August 2015^11^ and (b) 2675 patients enrolled in the Japanese Cardiac Registry of Heart Failure in Cardiology (JCARE‐CARD) who were hospitalized for heart failure at teaching hospitals from January 2004 to June 2005^14^ To comply with the SCD‐HeFT, eligible patients had New York Heart Association (NYHA) classification II or III and LVEF ≦35% after appropriate fluid management. The final propensity score matched cohort, with adjustments for age and NYHA classification, comprises 285 patients with defibrillator therapy and 285 patients with conventional therapy (Figure S1). The protocol for each research project had been approved by a suitably constituted Ethics Committee at each institution, and it conforms to the provisions of the Declaration of Helsinki.

### 2.2 Device programming

In general, device programming for patients with defibrillator therapy was as follows.^11^ VF zone detected ventricular events faster than 185–200 beats/min with at least one train of antitachycardia pacing (ATP) before shock, and the VT zone detected ventricular events faster than 150–170 beats/min with at least three trains of ATP before shock. After the multicenter automatic defibrillator implantation trial—reduce inappropriate therapy (MADIT‐RIT) trial was published in 2012,^15^ the VF zone ≧ 200–250 beats/min with ATP plus shock and VT zone ≧ 170 beats/min with delayed therapy (a 60‐second delay) or only monitoring were recommended. The discrimination algorithms, which discriminate between ventricular (VT) and supraventricular tachycardia (SVT), were used at the physician's discretion.

### 2.3 Outcomes

The analyzed events were (a) survival and (b) cause of death. In patients with defibrillator therapy, (c) appropriate defibrillator therapy and (d) complications associated with ICD/CRT‐D implantation were also evaluated.^11^, ^13^, ^14^ Appropriate defibrillator therapy was defined as antitachycardia pacing or shock for tachyarrhythmia determined to be either ventricular tachycardia (VT) or ventricular fibrillation (VF). The diagnosis of the cause of death was made by attending physicians.

### 2.4 Statistical analysis

All data are expressed as mean ± standard deviation (SD). To obtain the propensity score matched cohort, adjustments were made for age and NYHA classification with the caliper (SDs) of 0.2 (Figure S1). Simple between‐group analysis was conducted using Student's t‐test. Categorical variables were compared using the *χ* ^2^ test or Fisher's exact test. Kaplan–Meier curves were constructed to estimate event‐free outcomes in the study groups. Hazard ratios for outcomes were computed with a multivariate Cox proportional‐hazards regression model after adjusting for confounding factors such as gender, LVEF, and medications including angiotensin converting enzyme inhibitor (ACEI)/angiotensin II receptor blocker (ARB) and beta blocker. To evaluate the role of defibrillator therapy, we used cumulative incidence function (CIF) curves to estimate the probability of appropriate defibrillator therapy for first VT/VF, as the event of interest, and the probability of death without appropriate defibrillator therapy as a competing risk.^16^ The number of life‐days gained by ICD/CRT‐D implantation was also estimated using the restricted mean survival time measure. Differences with *p* < 0.05 were considered significant. EZR version 1.64^17^ (https://www.jichi.ac.jp/saitama‐sct/SaitamaHP.files/statmedEN.html accessed on January 28, 2024) was used for all statistical analyses.

## 3 RESULTS

### 3.1 Characteristics of heart failure patients with defibrillator therapy versus conventional therapy

Age was 64.6 ± 12.0 years in the defibrillator therapy group and 65.5 ± 12.4 years in the conventional therapy group. There was a significant difference with regard to gender, 82.8% male versus 74.0% male, between the defibrillator and conventional therapy groups (*p* = 0.014). Patients with the defibrillator therapy had a lower prevalence of comorbidities such as atrial fibrillation (AF), hyperuricemia, and cerebral infarction, and a higher prevalence of dyslipidemia as compared to those with the conventional therapy. LVEF of 25.6 ± 6.3% in the defibrillator group tended to be lower than that of 26.6 ± 6.0% in the conventional therapy group. The prevalence of the use of beta blockers and class III antiarrhythmic drugs was higher and that of ACEI/ARB was lower in the defibrillator therapy group than in the conventional therapy group. Other clinical characteristics and pharmacological therapies of the two groups are summarized in Tables 1 and 2.

**TABLE 1:** Characteristics of the patients.

	Defibrillator therapy group (*n* = 285)	Conventional therapy group (*n* = 285)	*p* value
Age (years)	64.6 ± 12.0	65.5 ± 12.4	0.40
Male	236 (82.8)	211 (74.0)	0.014
Underlying heart disease			1.0
Ischemic	104 (36.5)	104 (36.5)	
Nonischemic	181 (63.5)	181 (63.5)	
LVEF (%)	25.6 ± 6.3	26.6 ± 6.0	0.061
NYHA class			1.0
II	233 (81.8)	233 (81.8)	
III	52 (18.2)	52 (18.2)	
Heart rate (/min)	70 ± 15	71 ± 12	0.54
QRS duration (ms)	137.9 ± 36.0		
QT interval (ms)	445.9 ± 53.2		
Device			
ICD	105 (36.8)		
CRT‐D	180 (63.2)		
Atrial lead			
Absent	46 (16.1)		
Present	239 (83.9)		
AF	38 (13.3)	78 (27.4)	<0.0001
Diabetes mellitus	96 (33.7)	104 (36.5)	0.54
Hypertension^a^	114 (40.0)	113 (39.9)	1.0
Dyslipidemia^a^	93 (32.6)	69 (24.5)	0.033
Hyperuricemia^a^	55 (19.3)	143 (52.8)	<0.0001
Cerebral infarction^a^	21 (7.4)	36 (12.8)	0.036
BNP (pg/mL)^b^	651 ± 1641	647 ± 737	0.97
Hemoglobin (g/dL)^c^	13.0 ± 1.9	12.9 ± 2.3	0.63
Creatinine (mg/dL)^d^	1.40 ± 1.48	1.37 ± 1.15	0.82

*Note*: Values are means ± SD, or number (%).

Abbreviations: AF, atrial fibrillation; BNP, B‐type natriuretic peptide; CRT‐D, cardiac resynchronization therapy with a defibrillator; ICD, implantable cardioverter‐defibrillator; LVEF, left ventricular ejection fraction; NYHA, New York Heart Association.

^a^ Information regarding hypertension, dyslipidemia, hyperuricemia, and cerebral infarction in the conventional therapy group was missed in 2 patients, 3 patients, 14 patients, and 4 patients, respectively.

^b^ The value of BNP was missed in 29 patients in the defibrillator therapy group and 18 patients in the conventional therapy group.

^c^ The value of blood hemoglobin was missed in 4 patients in the defibrillator therapy group and 103 patients in the conventional therapy group.

^d^ The value of serum creatinine was missed in 8 patients in the defibrillator therapy group and 89 patients in the conventional therapy group.

**TABLE 2:** Pharmacological therapy.

	Defibrillator therapy group (*n* = 285)	Conventional therapy group (*n* = 285)	*p* value
Ia	1 (0.4)	0 (0.0)	1.0
Ib	12 (4.2)	25 (8.8)	0.028
Ic	3 (1.1)	5 (1.8)	0.50
Beta blockers	228 (80.0)	173 (61.1)	<0.0001
III	85 (29.8)	51 (18.0)	0.0011
Ca^2+^ antagonists	33 (11.6)	39 (13.8)	0.45
Digitalis	35 (12.3)	98 (34.6)	<0.0001
Diuretics	209 (73.3)	254 (89.8)	<0.0001
ACEI/ARB	205 (71.9)	236 (83.4)	0.0012
MRA	126 (44.2)	143 (50.5)	0.15
Nitrates	29 (10.2)	64 (22.6)	<0.0001
Statins	94 (33.0)	69 (24.4)	0.026
Oral anticoagulants	118 (41.4)	133 (47.0)	0.21
Antiplatelet drugs	133 (46.7)	134 (47.3)	0.93

*Note*: Data are given as number (%). With regard to pharmacological therapy, there were missing data in 2 patients in the conventional therapy group. Ia, Ib, Ic, and III indicates the class Ia, Ib, Ic, and III antiarrhythmic drug, respectively.

Abbreviations: ACEI, angiotensin converting enzyme inhibitor; ARB, angiotensin II receptor blocker; MRA, mineralocorticoid receptor antagonist.

### 3.2 Outcomes

**FIGURE 1:**
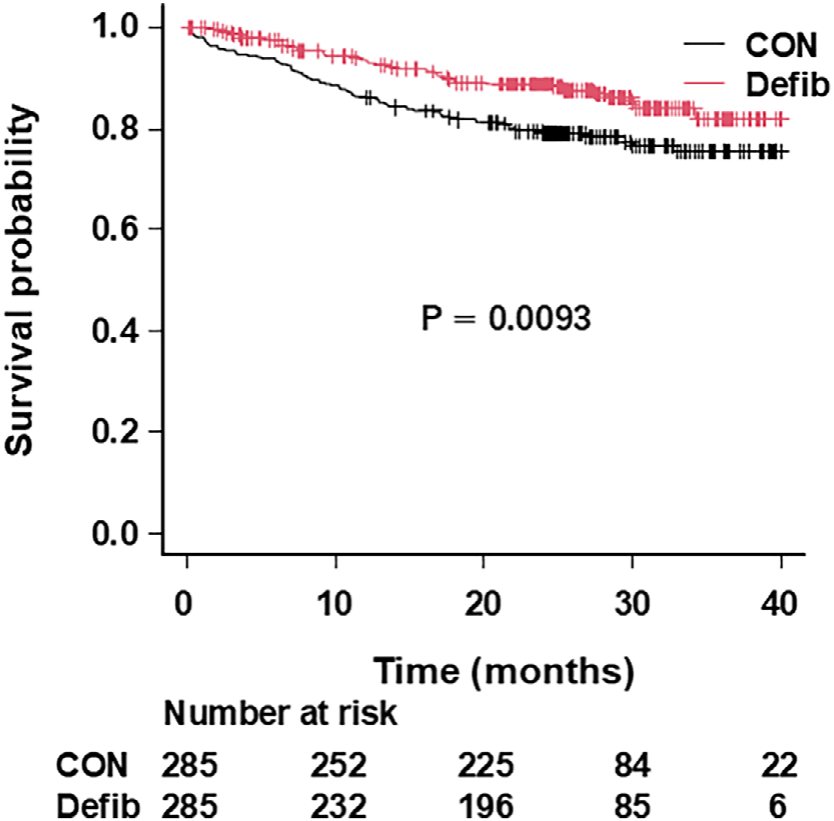
Cumulative survival free from all‐cause death in heart failure patients with or without a defibrillator. Cumulative survival probability is plotted for the defibrillator therapy group (Defib, red line) and conventional therapy group (CON, black line). The difference in survival between the two groups was significant (*p* = 0.0093, by the log‐rank test).

All‐cause death occurred in 35 of 285 patients with defibrillator therapy (12%) during an average follow‐up of 23 ± 11 months and 65 of 285 patients with conventional therapy (23%) during an average follow‐up of 25 ± 11 months. The survival rate was 93.4% at 1 year (95% confidence interval [CI]: 89.6–95.9), 88.7% at 2‐year (95% CI: 84.0–92.1) and 81.8% at 3‐year (95% CI: 74.1–87.5) in the defibrillator therapy group and 86.0% at 1‐year (95% CI: 81.4–89.5), 79.1% at 2‐year (95% CI: 73.9–83.4) and 75.4% at 3‐year (95% CI: 69.1–80.6) in the conventional therapy group (*p* = 0.0093) (Figure 1). Using the restricted mean survival time measure, the life gain was 74 days (95% CI: 23–126) at 3 years following ICD/CRT‐D implantation. There was also a significant difference with regard to the survival among three groups (*p* = 0.03), when the defibrillator therapy group was separated into the ICD and CRT‐D groups (Figure S2).

Sudden death occurred in 5 patients (14%: 5 of 35 all‐cause death) versus 21 patients (32%: 21 of 65 all‐cause death), heart failure death occurred in 10 patients (29%: 10 of 35 all‐cause death) versus 30 patients (46%: 30 of 65 all‐cause death), and noncardiac death occurred in 20 patients (57%: 20 of 35 all‐cause death) versus 14 patients (22%: 14 of 65 all‐cause death) in the defibrillator versus conventional therapy groups. The rate of all‐cause death, sudden death, heart failure death, and noncardiac death in the two groups, and hazard ratios for the respective events in the defibrillator versus conventional therapy group are summarized in Tables 3 and 4. There was a significant reduction in the risk of sudden death and heart failure death in the defibrillator therapy as compared to the conventional therapy group.

**TABLE 3:** Mortality rates in heart failure patients of the defibrillator and conventional therapy groups.

	At 1 year	At 2 years	At 3 years	*p* value
All‐cause death (%) (95% CI)				
Defibrillator therapy	6.6 (4.1–10.4)	11.3 (7.9–16.0)	18.2 (12.5–25.9)	0.0093
Conventional therapy	14.0 (10.5–18.6)	20.9 (16.6–26.1)	24.6 (19.4–30.9)	
Sudden death (%) (95% CI)				
Defibrillator therapy	0.8 (0.2–3.1)	2.1 (0.9–5.1)	2.1 (0.9–5.1)	0.0057
Conventional therapy	4.5 (2.6–7.7)	6.6 (4.1–10.4)	8.8 (5.4–14.3)	
Heart failure death (%) (95% CI)				
Defibrillator therapy	1.6 (0.6–4.2)	2.5 (1.1–5.5)	8.1 (3.8–16.9)	0.0030
Conventional therapy	6.9 (4.4–10.5)	11.2 (8.0–15.7)	11.2 (8.0–15.7)	
Noncardiac death (%) (95% CI)				
Defibrillator therapy	4.3 (2.4–7.7)	7.0 (4.4–11.1)	9.0 (5.9–13.8)	0.25
Conventional therapy	3.4 (1.8–6.4)	4.6 (2.6–8.0)	6.8 (3.8–12.0)	

Abbreviation: CI, confidence interval.

**TABLE 4:** Hazard ratios for events in heart failure patients in the defibrillator versus conventional therapy group.

Events	Hazard ratio	95% CI	*p* value
All‐cause death			
Unadjusted analysis	0.582	0.386–0.880	0.010
Adjusted analysis	0.616	0.402–0.943	0.026
Sudden death			
Unadjusted analysis	0.274	0.103–0.731	0.0097
Adjusted analysis	0.274	0.103–0.731	0.0097
Heart failure death			
Unadjusted analysis	0.355	0.173–0.726	0.0045
Adjusted analysis	0.362	0.172–0.764	0.0077
Noncardiac death			
Unadjusted analysis	1.49	0.753–2.952	0.25
Adjusted analysis	1.45	0.711–2.949	0.31

*Note*: Models were adjusted for the following covariates: LVEF, gender, and medications including ACEI/ARB and beta blockers.

Abbreviations: ACEI, angiotensin converting enzyme inhibitor; ARB, angiotensin II receptor blocker; CI, confidence interval; LVEF, left ventricular ejection fraction.

**FIGURE 2:**
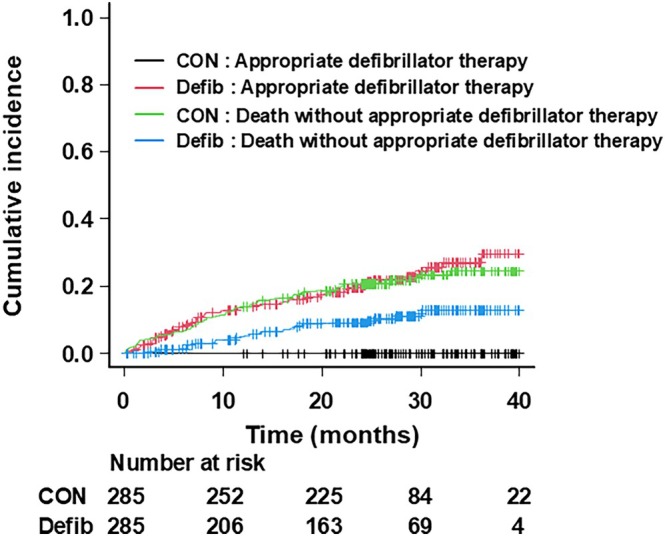
Cumulative incidence curve for appropriate defibrillator therapy and for the competing risk of death without appropriate defibrillator therapy in the defibrillator and conventional therapy group.

Figure 2 illustrates the CIF curves for the observed risk of first appropriate defibrillator therapy and for death without appropriate defibrillator therapy as a competing risk in the two groups. In the defibrillator therapy group, the observed risk of first appropriate defibrillator therapy at 1, 2, and 3 years was 14.0% (95% CI: 10.2–18.5), 19.8% (95% CI: 15.1–25.0), and 27.0% (95% CI: 20.7–33.8), which was higher than 5.1% (95% CI: 2.8–8.2), 9.3% (95% CI: 6.1–13.3), and 13.0% (95% CI: 8.7–18.2) of the corresponding risk of death without appropriate defibrillator therapy. Notably, the risk of death without appropriate defibrillator therapy in the conventional therapy group, which is the same as the rate of all‐cause death in this group, appeared to be similar to the observed risk of first appropriate defibrillator therapy in the defibrillator therapy group.

**FIGURE 3:**
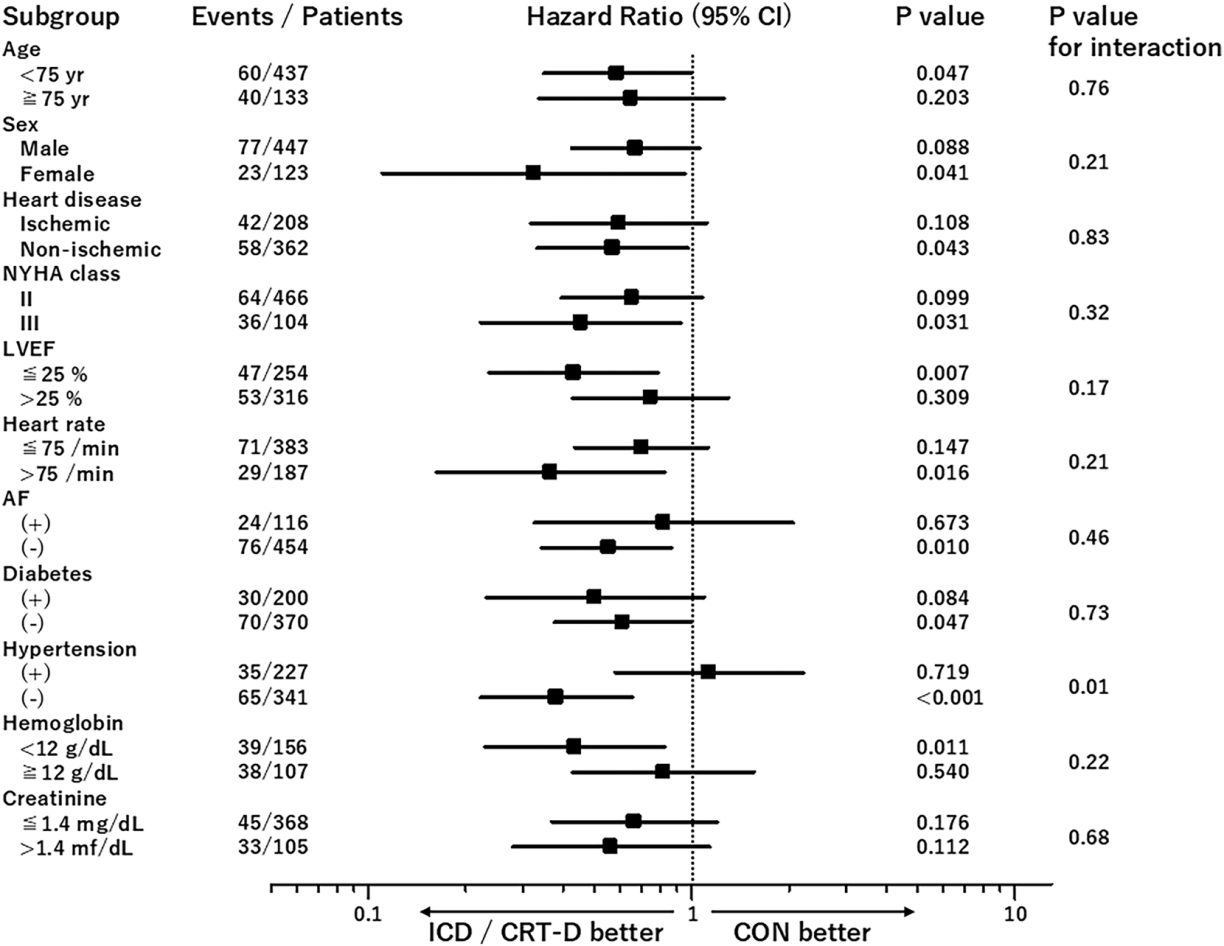
Forest plots showing hazard ratios and 95% confidence intervals for all‐cause death in the defibrillator versus conventional therapy group in different subgroups. The hazard ratios in the various subgroups were not statistically different except for hypertension versus no hypertension, with significant interaction (*p* = 0.01). (+): Present; (−): Absent. Abbreviations as in Table 1.

Figure 3 illustrates that improved survival associated with the defibrillator therapy was not present in patients with hypertension (*p* = 0.01 for interaction). There was no significant interaction between the defibrillator effect on survival and baseline characteristics stratified by age (≧75 years), gender, etiology of heart disease (ischemic vs. nonischemic), NYHA classification, LVEF (≦25%), heart rate (≦75/min), history of AF and diabetes, and level of blood hemoglobin (<12 g/dL) and serum creatinine (≦1.4 mg/dL).

### 3.3 Complications associated with the ICD/CRT‐D implantation

Two patients had major complications (hematoma and lead dislodgement) at the implantation. One patient underwent an unsuccessful coronary sinus lead implant. During the follow‐up, device infections occurred in three patients. The complication rate was 1.05% (95% CI: 0.23–3.05) at the implantation, 0.8% (95% CI: 0.2–3.1) at 1 year, 0.8% (95% CI: 0.2–3.1) at 2 years, and 1.7% (95% CI: 0.5–5.8) at 3 years (Table S1).

## 4 DISCUSSION

The present study represents the real‐world benefit of primary prevention ICD/CRT‐D implantation in HFrEF patients in Japan, who were eligible for the SCD‐HeFT. Hazard ratio (HR) of 0.616 for all‐cause death of the defibrillator versus conventional therapy (Table 4) was better than that of 0.77 in the SCD‐HeFT^7^ despite the average age being 5 years higher in our study. The effect of CRT may have contributed to the marked reduction of all‐cause death because the defibrillator therapy decreased the risk of not only sudden death but also heart failure death in our study. The latter was not observed in the SCD‐HeFT^18^ Moreover, the probability of appropriate defibrillator therapy in the defibrillator therapy group coincided with that of all‐cause mortality in the conventional therapy group (Figure 2). In the case of absence of the appropriate defibrillator therapies, sudden cardiac death might have occurred in patients in the defibrillator therapy group and have resulted in the CIF curve of all‐cause mortality being similar to that in the conventional therapy group, thereby reinforcing a beneficial effect of defibrillator therapy on primary prevention of arrhythmic death in HFrEF patients.

The contemporary multicenter cohort study (the EU‐CERT‐ICD Study), in which candidates for implantation of a CRT device were excluded, similarly demonstrated that primary prevention ICD implantation was associated with a 27% lower mortality (HR 0.731) in heart failure patients of LVEF ≦35% and narrow QRS.^19^ The subgroup analyses indicated a lack of ICD benefit in diabetics (adjusted HR = 0.945, *p* = 0.78, *P* for interaction = 0.089) or those aged ≧75 years (adjusted HR 1.063, *p* = 0.82, *P* for interaction = 0.090).^19^ In contrast, significant interaction was absent in diabetics and those aged ≧75 years, but was present in those with hypertension in our study (Figure 3). In the Multicenter Automatic Defibrillator Implantation Trial‐Cardiac Resynchronization Therapy (MADIT‐CRT) study, factors associated with a reduction in heart failure or death in response to CRT‐D as compared with ICD were (a) QRS duration ≧150 ms (HR = 0.53, *p* < 0.001, *P* for interaction = 0.04), (b) systolic blood pressure <115 mmHg (HR = 0.48, *p* < 0.001, *P* for interaction = 0.05) and (c) LBBB (HR = 0.47, *p* < 0.001, *P* for interaction = 0.002) for ischemic cardiomyopathy, and (a) females (HR = 0.25, *p* < 0.001, *P* for interaction = 0.001), (b) diabetics (HR = 0.31, *p* < 0.001, *P* for interaction = 0.05), and (c) LBBB (HR = 0.42, *p* < 0.001, *P* for interaction = 0.011) for nonischemic cardiomyopathy.^20^, ^21^ These interaction effects may partly explain the discrepancy between the EU‐CERT‐ICD and our study.

A controversy exists for ICD benefit in elderly patients. An age cutoff for ICD implantation at ≤70 years yielded the highest survival in the DANISH study because the ICD therapy was not associated with improved all‐cause mortality (HR 1.05, *p* = 0.84) in patients >70 years old.^22^ The I‐70 Study, a randomized clinical trial, tried to determine the efficacy of primary prevention ICD implantation in elderly (aged >70 years) veterans.^23^ With regard to all‐cause mortality, there was a trend favoring the ICD use over the first 36 months of follow‐up, whereas the recruited sample size was not enough to demonstrate the ICD effectiveness among elderly patients. In the post hoc analysis of the Heart Failure Indication and Sudden Cardiac Death Prevention Trial Japan (HINODE), the risk of all‐cause mortality was increased for the elderly (aged >70 years) versus nonelderly (aged ≦70 years) (HR 2.82, *p* = 0.039), but did not differ after excluding ICD patients with CRT‐D indication (HR 2.32, *p* = 0.11).^24^ Age cutoff alone is not likely to be a determinant to stratify the ICD benefit in Japan.

The number of victims of sudden cardiac death is increasing year by year, thereby reaching more than 91,000 cases in 2022. This number occupies 64% of the Utstein template for out‐of‐hospital cardiac arrest in Japan. Even if restricted to bystander‐witnessed cases, the rate of return to social activity with favorable outcomes (cerebral performance category score 1–2)^25^ was 7.5% (https://www.fdma.go.jp/publication/#rescue accessed on December 20, 2024). Accordingly, we unfortunately lost at least more than 84,000 cases from social activity because of sudden cardiac death or unfavorable neurological outcomes, which would cost a great amount of medical resources and result in a significant labor shortage. The loss of working resources can be critical because we are facing a rapid decrease in the Japanese population, probably leading to 95 million people with about 40% aged ≧65 years in 2050 (https://www.soumu.go.jp/main_content/000273900.pdf accessed on December 20, 2024). On the other hand, the number of de novo ICD/CRT‐D implantation (including both primary and secondary prevention) is almost steady from 6687 in year 2017 to 6384 in year 2023 (https://www.jadia.or.jp/medical/crt‐d.html accessed on December 20, 2024), which is far less than that of sudden cardiac death victims. With careful consideration of the indication, medical resources and cost effectiveness,^26^, ^27^, ^28^ appropriate ICD utilization for primary prevention of sudden cardiac death is required for the future.

There are some limitations to be considered in this study. First, the study populations were out‐of‐date, especially for the conventional therapy group, who were hospitalized for heart failure from January 2004 to June 2005. As rates of sudden death declined substantially over time among HFrEF patients probably because of a cumulative benefit of evidence‐based medications^29^ it is questionable whether these results can be applied to current patients, and we must interpret the present results with caution. Second, death without appropriate defibrillator therapy occurred in a substantial number of patients (Figure 2). The MADIT‐ICD benefit score and its modification^16^, ^30^ which assess the risk of VT/VF weighed against the risk of nonarrhythmic mortality, may better predict the likelihood of primary prevention ICD/CRT‐D benefit. In addition, we anticipate results of the PROFID project^31^ which may optimally identify high‐risk patients that can be protected from sudden cardiac death by ICD implantation with a personalized approach.

In conclusion, we have demonstrated that primary prevention ICD/CRT‐D reduced the risk of all‐cause mortality in Japanese patients eligible for the SCD‐HeFT criteria. Since primary prevention CRT‐D utilization is higher than ICD, with a rate of about 60% during 2011 to 2020 (Figure S3), the present study demonstrates the real‐world effectiveness of prophylactic defibrillator therapy for HFrEF patients in Japan.

## CONFLICT OF INTEREST STATEMENT

All authors declare no conflict of interest related to this study.

## ETHICS STATEMENT

The study was approved in the Ethics Committee of Sapporo City General Hospital on May 16, 2018 (Approval No.: H30‐057‐455).

## CONSENT

Patient consent has been obtained in an opt‐out manner in Sapporo City General Hospital.

## Supporting information


**Figure S1.** CONSORT (Consolidated Standards for Reporting Trials) diagram.
**Figure S2.** Cumulative survival free from all‐cause death in heart failure patients with or without a defibrillator.
**Figure S3.** Annual trends in number of patients with primary prevention ICD/CRT‐D implantation with the proportion of CRT‐D registered in the JCDTR and New JCDTR.
**Table S1.** Complication rates at the implantation and during the follow‐up in heart failure patients of the defibrillator therapy group.
