# Supplementary material for: Survival benefit of primary prevention implantable cardioverter‐defibrillator/cardiac resynchronization therapy with a defibrillator: Analysis of the Japan cardiac device treatment registry and Japanese cardiac registry of heart failure in cardiology
Source: J Arrhythm. 2025 May 12;41(3):e70084. doi: 10.1002/joa3.70084 (PMC12067054; doi:10.1002/joa3.70084)
Supplement: Supplementary file 1 — Figure S1. CONSORT (Consolidated Standards for Reporting Trials) diagram. Figure S2. Cumulative survival free from all‐cause death in heart failure patients with or without a defibrillator. Figure S3. Annual trends in number of patients with primary prevention ICD/CRT‐D implantation with the proportion of CRT‐D registered in the JCDTR and New JCDTR. Table S1. Complication rates at the implantation and during the follow‐up in heart failure patients of the defibrillator therapy group. [file JOA3-41-e70084-s001.docx]

**Supplemental Figure**

**Figure S1. CONSORT (Consolidated Standards for Reporting Trials) diagram.**

**Figure S2. Cumulative survival free from all-cause death in heart failure patients with or without a defibrillator.**

Cumulative survival probability is plotted for the CRT-D therapy group (CRT-D, red line), ICD therapy group (ICD, green line) and conventional therapy group (CON, black line). The difference in survival among the three groups was significant (P=0.03, by the log-rank test adjusted with Bonferroni method).

**Figure S3. Annual trends in number of patients with primary prevention ICD / CRT-D implantation with the proportion of CRT-D registered in the JCDTR and New JCDTR.**

Absolute number of patients with primary prevention CRT-D and ICD implantation is given by orange bars and blue bars, respectively. A dark blue line indicates the proportion of CRT-D among primary prevention ICD / CRT-D.

This figure was made using the data from Yokoshiki H, et al, J Arrhythm 2023;39:757-765.

**Figure S1**


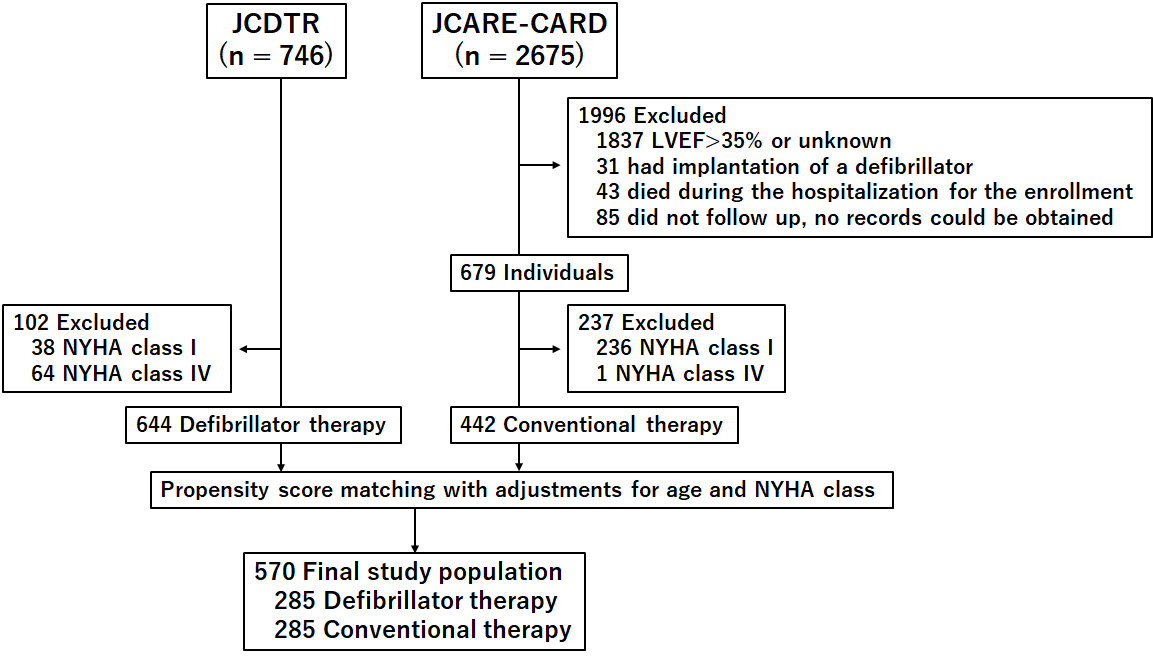


**Figure S2**


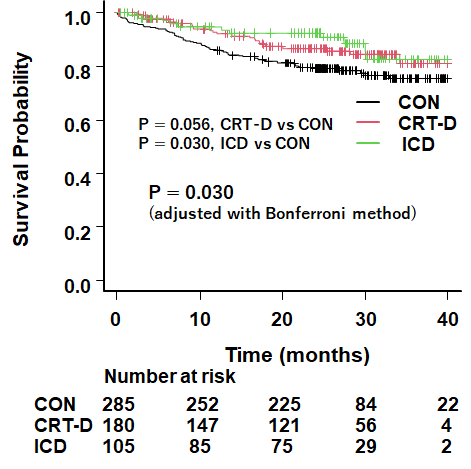


**Figure S3**


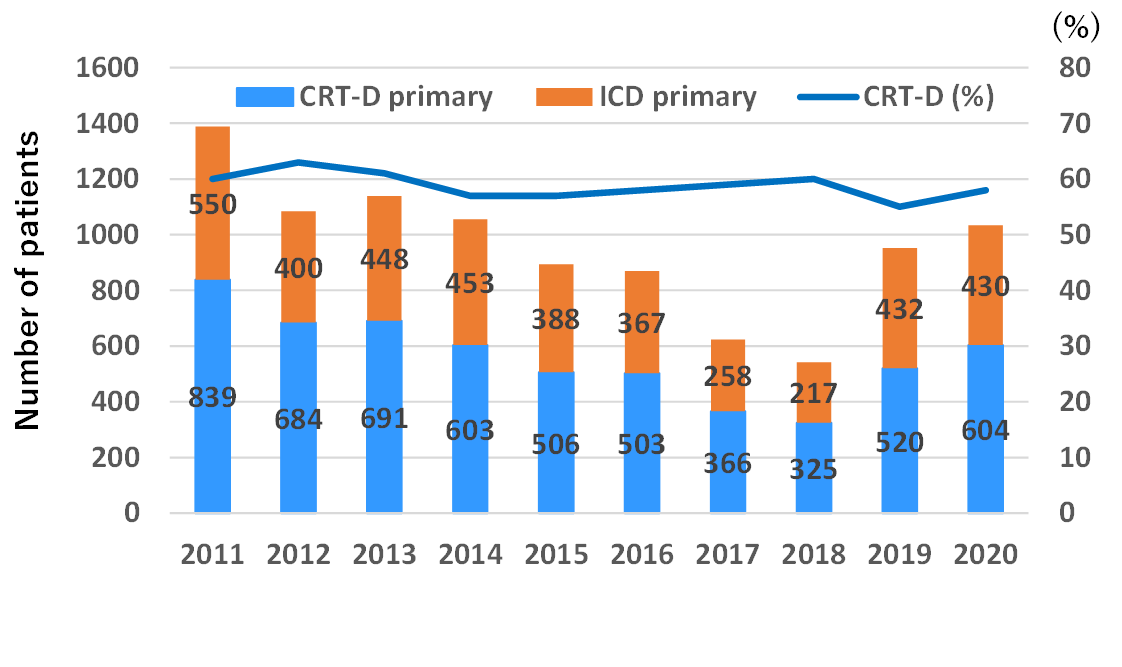


**Supplemental Table. Complication rates at the implantation and during the follow-up in heart failure patients of the defibrillator therapy group**

| At the implantation | At 1 year | At 2 years | At 3 years |
| --- | --- | --- | --- |
| 3 (1.05, [0.23 – 3.05]) | 2 (0.8, [0.2 – 3.1]) | 0 (0.8, [0.2 – 3.1]) | 1 (1.7, [0.5 – 5.8]) |
| Detailed breakdown | | | |
| Hematoma 1 | Infection 2 |  | Infection 1 |
| Lead dislodge 1 |  |  |  |
| CS lead implant failure 1 |  |  |  |

Data are given as number (%, [95%CI]).

CI: confidence interval; CS: coronary sinus.
